# Minimal Effects of Rearing Enrichments on Pullet Behaviour and Welfare

**DOI:** 10.3390/ani10020314

**Published:** 2020-02-18

**Authors:** Dana L.M. Campbell, Priscilla F. Gerber, Jeff A. Downing, Caroline Lee

**Affiliations:** 1 Agriculture and Food, Commonwealth Scientific and Industrial Research Organisation (CSIRO), Armidale, NSW 2350, Australia; caroline.lee@csiro.au; 2 School of Environmental and Rural Science, University of New England, Armidale, NSW 2351, Australia; pgerber2@une.edu.au; 3 School of Life and Environmental Science, Faculty of Veterinary Science, University of Sydney, Sydney, NSW 2006, Australia; jeff.downing@sydney.edu.au

**Keywords:** behaviour, manual restraint, novel objects, organ weights, coping style, adrenal, chicken, CELL-DYN 3700

## Abstract

**Simple Summary:**

Free-range pullets are often reared indoors, which may make it difficult to adapt to being outside as adult hens. Enrichments during rearing could improve the birds’ behavioural and physical development. Hy-Line Brown^®^ chicks (*n* = 1700) were reared indoors across 16 weeks with 3 enrichment treatments: (1) a standard control; (2) novel objects provided weekly (novelty) or (3) perching structures (structural) provided. All pullets were weighed at 5, 8, 12, and 16 weeks old. Pullets were also tested in two behavioural tests at 9 (*n* = 87) and 16 (*n* = 90) weeks of age, assessing fear and responses to stress. At 15 weeks, lymphoid organs were extracted and weighed from 90 pullets. Pullets were transferred to the free-range facility at 16 weeks and housed in 9 identical pens within rearing treatments. Hens perching were counted via video recordings across the first week. Structural hens perched less than the novelty hens in the layer facility (*p* = 0.02) but there were few other consistent rearing treatment differences. The rearing environments had minimal effects on pullet behaviour and welfare; greater differences may be seen in the adult hens.

**Abstract:**

In Australia, free-range pullets are typically reared indoors, which may hinder later adjustment to outdoor access. Rearing enrichments could optimise pullet development. Hy-Line Brown^®^ chicks (*n* = 1700) were reared indoors across 16 weeks with 3 enrichment treatments: (1) a standard control; (2) novel objects (novelty) provided weekly or (3) perching structures (structural) provided. All pullets were weighed at 5, 8, 12, and 16 weeks old. Pullets (*n* = 87) were tested in a novel arena at 9 weeks and manual restraint (*n* = 90) at 16 weeks. At 15 weeks, lymphoid organs were extracted and weighed from 90 pullets. Pullets were transferred to the free-range facility at 16 weeks and housed in 9 identical pens within rearing treatments. Hens perching were counted via video recordings across the first week. The structural pullets had the highest relative adrenal weights (*p* = 0.03) but differences may not have been biologically relevant. Structural hens perched less than the novelty hens in the layer facility (*p* = 0.02). There were no other consistent rearing treatment differences. The rearing environments had minimal effects on pullet behaviour and welfare, but data from the adult hens did show some longer-term welfare impacts.

## 1. Introduction

Rearing environments for laying hens are important for the physical and behavioural development of pullets [1]. Providing resources in the rearing environment such as litter substrate or perches can ensure appropriate behavioural patterns are formed [2], physical strength is enhanced [3], or immune function is strengthened [4,5], thereby optimising hen welfare as adults. It is particularly important to consider how the rearing conditions may affect adaptability to a specific type of layer housing system, with recommended system matching between the rearing and adult phases [6]. For example, rearing pullets in aviaries will make them better placed to perform in a layer aviary system and be competent in navigating the different resource areas, particularly when first transferred [7]. This system matching can be more challenging to achieve for free-range hens. Within Australia (and elsewhere), pullets destined for free-range systems are typically reared indoors due to the health concerns of exposing unvaccinated birds to outdoor pathogens and the logistics of the current rearing house designs (they do not have outdoor access). This may impact the ability of the hens to adapt to the outdoor environment as adults, where they will be exposed to new experiences such as sunlight, weather variation, predators, and management inconsistencies (e.g., closing of range access pop-holes during inclement weather). In the absence of feasible rearing options where pullets may gradually get exposed to the outdoor environment to improve their willingness to access it [8], provision of enrichments during the rearing period for these pullets may be an alternative method of optimising their development. 

Enrichments have been defined as environmental modifications that improve an animal’s biological functioning [9]. For laying hens, there are a diverse range of enrichments if the baseline comparison is a conventional cage or floor-rearing. Rearing enrichments could include perches, structures, and additional objects or stimulatory playbacks (e.g., auditory) which may or may not be supplementary to a litter substrate. Previous research with enrichment provision has demonstrated an array of behavioural and welfare impacts on the chicks/pullets and during the transition period to the layer house, but not in all cases. Classical music played to chicks reduced their heterophil/lymphocyte (H/L) ratio and skeletal fluctuating asymmetry relative to chicks reared with no music, indicating stress-reducing effects of this form of enrichment [10]. However, auditory enrichment showed no consistent reduction to the duration of tonic immobility (fear response), and physical enrichments in the form of pecking strings and barley grains did not impact any of these behavioural and welfare measures [10]. Similarly, Hartcher and colleagues [11] found minimal impacts of enrichment with pecking strings, litter, and scattered oats on pullet responses in open field and tonic immobility tests. Aviary-rearing compared with cage-rearing reduced fearful responses toward novel objects in the first few weeks following transfer to a furnished cage system [12] and increased the use of the three-dimensional area in the experimental laying house [13]. However, there were no differences in faecal corticosterone metabolites, and the differences in system use were equivalent between rearing groups after four weeks in the new housing environment [13]. Comparisons between pullets reared in standard cages or cages with perches, strings, a dust bathing area and a nest box showed that these rearing enrichments improved immune responses under an imposed social stressor, including lowered H/L ratio and increased antibody production [4]. Similar enrichment resources also modified non-specific immune system responses following transport stress relative to non-enriched birds [5] and affected the weights of lymphoid organs [4,5], indicating the physiological impacts of varying developmental environments. With increasing recognition of the importance of layer rearing environments and consumer concerns for improved layer welfare, further evidence is needed for the potential benefits of rearing enrichments, particularly for free-range hens that experience large changes between rearing and layer systems. 

The objectives of this study were to assess the impacts of different types of enriched rearing environments on the fear and coping style responses of pullets during behavioural tests, their physical health, and their behavioural adaptation to the laying facility following transfer. We predicted that varying types of enrichment would reduce fear and stress-related responses and improve pullet physical health and adaptation in comparison to control (non-enriched) birds. 

## 2. Materials and Methods 

### 2.1. Ethical Statement

All research was approved by the University of New England Animal Ethics Committee (AEC17-092).

### 2.2. Animals and Pullet Housing

The study was conducted in the Kirby Poultry facility of the University of New England, Armidale, Australia. A total of 1700 Hy-Line^®^ (West Des Moines, Iowa, USA) Brown day-old layer chicks were housed within 9 floor pens (6.2 mL × 3.2 mW) across three separate rooms. Each pen contained rice hulls as a litter substrate, 15 water nipples (approximately 12 birds/nipple), and 4 round feeders (1.2 m circumference each, approximately 2.5 cm/pullet), provided in accordance with the current Australian Model Code of Practice for the Welfare of Animals-Domestic Poultry [14]. Water and commercially mixed mash formulated for specific growth stages was provided ad libitum. The pullets were reared for 16 weeks at this facility and exposed to three different rearing treatments, with a treatment replicate in each room, balanced for location. The enrichment treatments were: (1) a control treatment with no additional enrichments, (2) a novelty treatment (various novel objects were added/removed approximately weekly including balls, bottles, brooms, buckets, disks, ropes, chain, cinder blocks, containers, dog toys, milk jugs, plastic kids toys, pipes and strings), and (3) a structural treatment. This structural treatment included four custom-designed perching apparatuses constructed of metal, painted black, and coated in a non-slip covering (L, W, H = 0.60 m). They had two solid sides and one open-framed side, forming an H-shaped structure that could be placed in different orientations. Pullets could perch on these, but the solid side in some orientations would provide a visual/physical obstruction requiring the birds to navigate around it. Space was available underneath the structures for equal floor density in all treatments. Shade cloth was hung on the wire pen dividers to visually isolate birds and enrichments. At 16 weeks of age, bird density was approximately 15 kg/m^2^, or 9 pullets/m^2^, with a total of 174–190 birds per pen (pen variation resulted from both chick mortality and some placement error). The temperature and lighting schedules followed the Hy-Line^®^ Brown recommendations for alternative system management [15]. However, the artificial LED lighting (no natural light) was maintained at 100 lux because the pullets were destined for an outdoor environment. The rooms were mechanically ventilated with heating as needed but no cooling system. The chicks and pullets were vaccinated to meet both regulatory requirements and standard recommendations for the region (Newcastle disease, Marek’s disease, fowl pox, fowl cholera, egg drop syndrome, *Mycoplasma gallisepticum*, *Mycoplasma synoviae*, infectious bronchitis, infectious laryngotracheitis, and avian encephalomyelitis). 

### 2.3. Data Collection Procedures

#### 2.3.1. Body Weight

All birds were assessed for body weight at 5, 8, 12, and 16 weeks of age. At 5 weeks of age, birds were weighed in crates to get an average chick weight (~40 chicks/crate). From 8 weeks onwards, all pullets were of sufficient size to be individually weighed using poultry-specific electronic hanging scales (BAT1; VEIT Electronics, Moravany, Czech Republic). 

#### 2.3.2. Novel Arena Test

**Figure 1:**
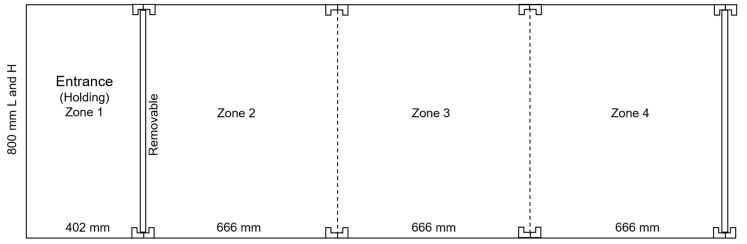
The layout and dimensions of the novel arena used to test pullets, with the holding zone (zone 1) and other zones (2–4) indicated.

At 9 weeks of age, 87 pullets (*n* = 29 per rearing treatment) were randomly selected from their home pens (the selection was balanced across treatment pen replicates and rooms) and tested in a novel arena (Figure 1). All testing was conducted in a separate room at the same facility. The wooden novel arena was a rectangle (2.4 m × 0.8 m × 0.8 m) that was placed on a cement floor (no substrate present) and covered with wire mesh to prevent the birds escaping. The rectangle was divided into four zones by metal framework (that was laid flat on the floor and held the wire mesh roof in place), with the first zone having a sliding door that could hold the birds in that area. The holding zone (1) was 42 cm and zones 2 to 4 were each 67 cm in length. An individual bird was caught from the home pen and carried to the test room with a towel over their head to shield their eyes and induce calmness (they had to be carried outdoors to enter the testing room). They were placed in the holding area (zone 1) for twenty seconds before the removable wooden separator door was slid open. The bird was free to move within the arena for a 10-min period before being returned to the home pen. Pullets were leg-banded with coloured and numbered rings after their trial to ensure they could be excluded from subsequent behavioural testing and sampling. All experimenters were out of sight but within the room during testing. The testing order was balanced across pens and treatments, and occurred across a 3-day period from 08:00 until 18:00. The behaviour of each bird was video-recorded (Sony HDR-PJ410 Handycam, Sony Corporation, Tokyo, Japan) and later observed by a single experimenter blinded to the rearing treatment using The Observer XT 12.0 software (Noldus Information Technology, Wageningen, The Netherlands). The experimenter recorded the time that each bird spent in each zone, where zone 1 was the placement/holding zone, and zone 4 was at the other end of the arena. 

#### 2.3.3. Manual Restraint

At 16 weeks of age, 90 pullets (30 per treatment) were randomly selected (excluding arena-tested pullets) from each pen (10 per pen replicate) across a period of two days. Pullets were taken into one of two separate enclosed rooms adjacent to the home pens and immediately manually restrained by holding the bird in a right lateral recumbent position on a table for 5 min. The experimenter’s right hand held the hen’s side while the left hand gently stretched the legs. A second experimenter sitting at the table recorded the latency to first struggle (i.e., the hen attempting to pull their legs back up), latency to first vocalise, and number of vocalisations. The number of struggles was counted by the experimenter holding the bird. All testing was video-recorded (Sony HDR-PJ410 Handycam, Sony Corporation, Tokyo, Japan) with latency and count data later cross-checked for accuracy, and any errors made during live data recording were corrected. All experimenters were blind to the rearing treatments; pullet selection per pen replicate and treatment was balanced across the day. Following completion of the test, pullets were placed in crates in an enclosed outdoor tent to allow the corticosterone response to peak. Fifteen minutes after the start of their test, a 2 mL blood sample was collected from the brachial vein. The blood samples were collected in EDTA-coated tubes, centrifuged at 700 × g for 15 minutes on the day of collection to extract plasma, and plasma was stored at −20 °C until the radioimmunoassay was carried out. Plasma corticosterone concentrations were measured via the protocols as per [16]. Following the blood sample, the pullets were leg-banded with coloured and numbered rings and returned to their home pen. 

#### 2.3.4. Organ Weights

At 15 weeks of age, 90 randomly selected pullets (excluding behaviourally tested pullets) across the three rearing treatments (30 per treatment, 10 per pen replicate) were weighed, killed via cervical dislocation, and dissected to remove the liver (and gallbladder), bursa of Fabricius, spleen, thymus, and adrenals. These organs were weighed immediately following removal to the nearest 0.001 g. All dissections occurred across one day with pullets from treatments and pen replicates balanced across time, and all personnel collecting data were blind to the rearing treatments. 

#### 2.3.5. Blood Samples

One week prior to transport to the layer facility, 2 mL of blood samples were taken from the brachial veins of 90 randomly selected 15-week-old pullets (excluding behaviourally tested pullets) across all treatments (30 per treatment, 10 per pen replicate). The blood was sampled into EDTA-coated tubes, stored on ice, and processed using the CELL-DYN 3700 analyser veterinary package (Abbott, Abbott Park, IL, USA) according to the manufacturer’s instructions to determine blood cell types, including the concentrations of heterophils and lymphocytes and the heterophil/lymphocyte ratio. The pullets were identified with a coloured and numbered leg band and the same birds were sampled again at 16 weeks, the morning after the previous day’s weighing and transport to the layer facility.

#### 2.3.6. Layer Housing and Video Recording

**Figure 2:**
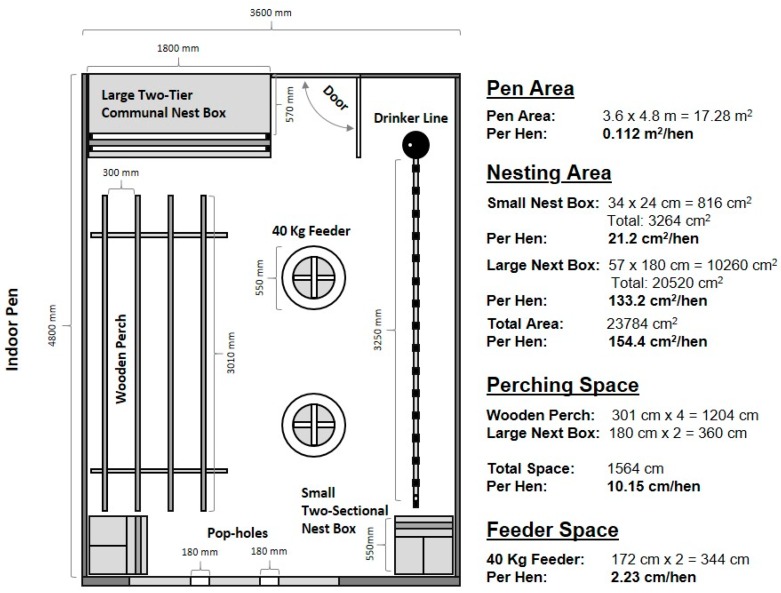
The layout and dimensions of one indoor pen at the layer facility showing the placement and dimensions of all resources. The resource allocation per hen is also indicated. Each pen was of the same set-up except three of the nine pens (balanced across treatments), contained a radio-frequency identification component box that one of the small nest boxes was placed upon.

At 16 weeks of age, all remaining pullets (*n* = 1386, some pullets were rehomed as they were additional to the capacity of the layer shed) were weighed and then placed into crates and transported (8.1 km) to the layer facility. Pullets were in crates for up to 2 h total, while all birds in a pen were weighed, transported, and unloaded across the day. The layer facility had 9 identical pens (4.8 mL × 3.6 m W, Figure 2) in a single shed with shade cloth on the wire pen dividers to visually isolate the birds. The pullets within the three treatment replicates were socially remixed, but each pen contained pullets from only one rearing treatment. Each pen had two suspended round feeders, 15 water nipples (single water line), perches, and nest boxes with rice hulls as the floor substrate. The perches were racks with four tiers (301 cm in length), and the large two-tiered nest boxes had perches at each tier that were 57 cm and 97 cm off the ground. Available nest box space, perches, feeder and water resources were provided to meet the Australian Model Code of Practice for the Welfare of Animals—Domestic Poultry, Figure 2 [14]. The perching space was 10 cm per bird due to logistical space restrictions within the pen, but birds also perched on the tops of the feeders and waterlines. Commercial pre-lay mash was provided ad libitum. The LED lighting schedule was at 10 hours light and 14 hours dark (changed to 11 h L: 13 h D at 17 weeks of age) with mean light intensity at 10.0 (± 0.84 SE) lux (Lutron Light Meter, LX-112850; Lutron Electronic Enterprise CO., Ltd, Taipei, Taiwan), as measured at the birds’ eye height from 3 locations (front, middle, back) within each pen (the brightest bulbs allowed by the shed wiring system). The shed was fan-ventilated with no other environmental controls.

Hikvision Network video cameras (Model DS-2CD2232-I5 4 mm, Hikvision Australia, Mt Waverly, VIC) were installed in each pen to record the pen area for the first 6 days following placement during the light period, and a portion of the dark period before lights came on/off (lights were on from 06:15 to 16:15 at 16 weeks, and from 06:15 to 17:15 at 17 weeks). The video was later observed by a single observer (blinded to rearing treatment), who counted the number of birds perched in different areas of the pen (perches, nest box perches, tops of feeders, waterline) every 30 min from 06:00 until 18:00 (point counts were made). 

### 2.4. Data and Statistical Analyses

All analyses were conducted in JMP 14.0 (SAS Institute, Cary, NC) with α set at 0.05. The crate body weight data from 5 weeks of age were compiled per pen (4 to 5 crates weighed per pen) and analysed using a General Linear Mixed Model (GLMM), with rearing treatment as a fixed effect and pen nested within rearing treatment as a random effect. Body weight data from 8, 12, and 16 weeks were compiled per individual bird and a GLMM was applied to compare the fixed effects of rearing treatment, age, and their interaction with pen nested within rearing treatment as a random effect. Tukey’s HSD tests were applied to the least squares means where significant differences were present. 

The time spent in each of the four zones in the novel arena test was converted to proportions of total test time for each bird and logit-transformed. A constant of 0.001 was first added to the proportional data to include the data when pullets did not enter a zone (value of zero) in the analyses. Seventeen datapoints (of 364) were not included in the analyses as the proportional value was 1 (total time spent in zone 1) and they were unable to be transformed. The data were analysed using a GLMM with zone, and zone x treatment as fixed effects and bird ID nested within pen and rearing treatment as a random effect. Tukey’s HSD tests were applied to the least squares means where significant differences were present. 

The organ weight data for 89 birds (data missing for one bird) were converted to a relative proportion of live body weight, compiled per bird and logit-transformed. GLMMs were applied to analyse the fixed effect of rearing treatment with bird ID nested within pen and rearing treatment as a random effect. Tukey’s HSD tests were applied to the least squares means where significant differences were present. 

The latencies to first struggle and vocalise in the manual restraint test were analysed using separate Kaplan—Meier estimates with a log-rank test for differences between enrichment treatment groups. The counts of struggles and vocalisations were square-root transformed. The number of struggles, vocalisations, and plasma corticosterone concentrations per bird were analysed using GLMMs with rearing treatment as a fixed effect and bird ID nested within pen and rearing treatment as a random effect. 

The concentrations of heterophils and lymphocytes and the H/L ratio from the CELL-DYN analyses were compiled per bird before and after transport and analysed using a GLMM with rearing treatment and transport as fixed effects and bird ID nested within pen and rearing treatment as a random effect. Tukey’s HSD tests were applied to the least squares means where significant differences were present.

Video counts of birds perching in all observed locations were summed to a single total per day of observation for each pen of pullets (*n* = 54, 9 pens × 6 days) and square-root transformed. A GLMM was applied with rearing treatment as a fixed effect, and pen nested within rearing treatment and day as two random effects. Tukey’s HSD tests were applied to the least squares means where significant differences were present.

## 3. Results

There were no differences between rearing treatments in chick body weight at 5 weeks of age (F_(2,6)_ = 0.12, *p* = 0.89). There was a significant interaction between age and rearing treatment for body weight from 8 to 16 weeks (F_(4,4927)_ = 3.34, *p* = 0.01). At eight weeks of age, the novelty pullets were of lower body weight compared with the structural pullets, but neither differed from the control pullets (Table 1). At each age point, the body weights were within or exceeded the recommended pullet body weight range [15]. 

There was no significant effect of rearing treatments on the proportion of time that the pullets spent in the four zones within the novel arena (F_(6,232.9)_ = 0.81, *p* = 0.56), but all pullets did spend the greatest time in zone 1 and the least in zone 4 (F_(3,232.2)_ = 28.11, *p* < 0.0001, LSM ± SEM raw proportional data; zone 1: 0.52 ± 0.03, zone 2: 0.22 ± 0.03, zone 3: 0.17 ± 0.03, zone 4: 0.09 ± 0.03). 

There was no effect of rearing treatment on the relative weights of the liver (F_(2,86)_ = 1.0, *p* = 0.37), spleen (F_(2, 86)_ = 0.59, *p* = 0.56), bursa (F_(2,86)_ = 0.65, *p* = 0.52), or thymus (F_(2,86)_ = 0.42, *p* = 0.66). There was a significant difference in the relative weight of the adrenal glands (F_(2,85)_ = 3.51, *p* = 0.03), with the structural pullets having larger adrenal glands than the control or novelty pullets (Table 2).

There were no differences between rearing treatment groups in the latency to first struggle (χ^2^ = 1.32, df = 2, *p* = 0.52) or first vocalise (χ^2^ = 1.75, df = 2, *p* = 0.42) during manual restraint testing (Table 3). There were also no differences in the total number of struggles (F_(2,87)_ = 0.60, *p* = 0.55), total number of vocalisations (F_(2,87)_ = 0.80, *p* = 0.45), or the plasma corticosterone concentrations (F_(2,87)_ = 0.86, *p* = 0.43, Table 3).

There was a significant interaction between rearing treatment and transport on the H/L ratio (F_(2,87)_ = 4.15, *p* = 0.02), with the structural hens showing a lower ratio following transport, while the control and novelty groups did not show a significant change (Table 4). There was no effect of rearing treatment (F_(2,87)_ = 0.42, *p* = 0.66), transport (F_(1,87)_ = 0.45), *p* = 0.50) or their interaction (F_(2,87)_ = 2.43, *p* = 0.09) on the concentrations of heterophils (Table 4). There was a significant effect of transport (F_(1,87)_ = 4.85, *p* = 0.03) on the concentrations of lymphocytes, with an increase after transport (Table 4), but there was no effect of rearing treatment (F_(2,87)_ = 1.68, *p* = 0.19) and no interaction between transport and rearing treatment (F_(2,87)_ = 1.08, *p* = 0.35, Table 4). However, the concentrations of lymphocytes and heterophils were outside of the expected normal range for adult domesticated fowl (*Gallus domesticus*) as reported in [17]: lymphocytes: reported normal range 1.2–4.2 × 10^9^/L; this study: 1.4–55.01 × 10^9^/L; heterophils: reported normal range 0.5–7.6 × 10^9^/L; this study: 4.9–19.0 × 10^9^/L. The CELL-DYN 3700 also categorised the total number of white blood cells, monocytes, eosinophils, basophils, total red blood cells, hemoglobin, hematocrit, mean corpuscular volume (MCV), mean corpuscular hemoglobin (MCH), mean corpuscular hemoglobin concentration (MCHC), and platelets. Some of these parameters were also outside of the normal reported range for chickens (e.g., MCH: reported normal range 32.0–43.9 pg; this study: 46.67–64.59 pg, MCHC: reported normal range 30.2–36.2 g/DL; this study: 44.81–63.85 g/DL). 

**Figure 3:**
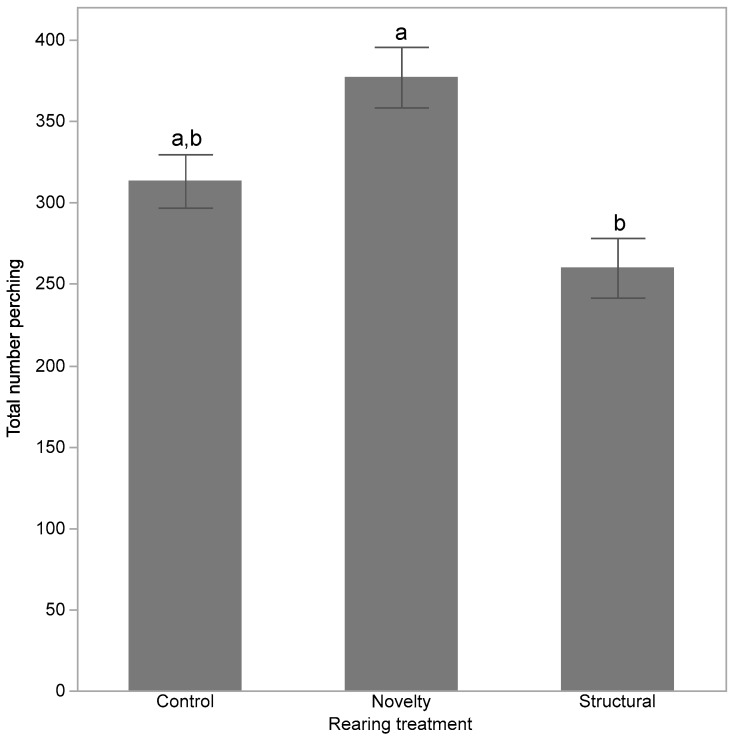
The total number of birds observed perching within the pen across the day for pullets exposed to three rearing enrichment treatments (control, novelty, structural). ^a,b^ Dissimilar letters indicate significant differences between rearing treatments.

There were significant differences between rearing treatments in the number of pullets that were observed perching within the pen in the first 6 days following transfer (F_(2,6)_ = 7.62, *p* = 0.02). The novelty birds perched more than the structural birds but neither group differed from the control (Figure 3). 

## 4. Discussion

This study researched the behavioural and physical impacts of different rearing enrichments on the development of pullets, but limited effects were found. Despite the variation in the chick rearing environments, the measurements taken on body weight, organ weights, fearfulness, coping style, and perching in the layer facility showed minimal differences. There were some effects of enrichments on body weight at one age point only, adrenal weights were highest in the structural pullets, and perching following transfer was higher in the novelty over structural hens. There were also some effects on blood parameters, but the accuracy of the CELL-DYN 3700 is questionable. Overall, no clear patterns emerged, but this may have been a result of the early age of the birds, with impacts of rearing enrichments becoming more distinct as the hens aged. 

The novel arena test showed no differences based on rearing treatments but all pullets showed high fear responses, spending around 50% of their time in the first zone that they were placed in and only 9% of their time at the far end of the arena. We predicted that the pullets exposed to the novel objects, in particular, would be more adaptable when placed in a novel environment, but this was not shown by the measures taken in this test. It may have been that at their young age, all pullets were at a maximum level of fear, masking any potential impacts of the enrichments in reducing fear. Previous research showed no differences in open field test behaviour at 13 weeks of age between pullets reared with strings, oats, and deep litter and pullets reared on shallow litter only [11], although the enriched pullets did show a longer latency to first step when tested earlier at five weeks of age [11]. Fear-related behavioural tests conducted across age in different layer strains did indicate high levels of fear at around 12 to 13 weeks of age [18]. Comparatively, novel object enrichments and wall drawings provided to chicks reduced fear responses in a range of behavioural tests that were applied in the first two to three weeks of age [19]. Testing on the birds at a later age may have revealed differences, particularly following the behavioural changes associated with sexual maturity [20]. The anecdotal observations of the birds in the current experiment showed that the pullets would actively avoid human contact, but the sexually matured hens would preferably approach personnel. 

There were also no differences found in the manual restraint test. Compared to previous manual restraint tests conducted in the same manner on adult hens around 38 weeks of age [21], the 16-week-old pullets showed a much shorter latency to vocalise, more vocalisations, a longer latency to struggle with fewer struggles, and a higher corticosterone response. In terms of coping style, which categorises the way animals respond to stressful situations [22], the reduced number of struggles and higher corticosterone responses in the pullets (relative to the older hens) would categorise all the pullets as ‘reactive’ rather than ‘proactive’; a more flexible behavioural response strategy [22]. However, these pullets made a much higher number of vocalisations compared to the adult hens, which could be indicative of a proactive style. Similar to other applications of the manual restraint test in layers, the results are not always consistent in categorising hens to a specific coping style [23], or consistent between studies (e.g., on feather pecking lines [24,25]). Different tests that measure coping style or personality traits may have distinguished between the pullets [26]. Alternatively, similar to the novel arena tests, the enriched environments may not have been enough to modify the behaviour of the pullets if they all found the manual restraint test highly stressful at the younger age. 

In contrast with previous literature, the pullets reared with structures failed to show the greatest use of the layer facility perches and other features requiring hens to jump following transfer. It was expected that pullets reared with structures that facilitated perching would develop the muscles and skills necessary to navigate the pen resources following transfer as has been demonstrated in several previous studies [7,27,28]. However, the structural hens perched less than the novelty treatment but not the control hens. Although no specific counts were made during rearing, the structural pullets were observed to use their provided enrichments starting at around two weeks of age. The novelty hens were initially provided enrichments that they were also able to jump onto, such as overturned containers, which may have developed their perching skills within the first two weeks, as perching starts from one week of age [29]. Unavoidably, pullets across all treatments also perched on the waterlines and tops of feeders so the control pullets were not completely devoid of perching experience. The implemented structures may not have been of comparatively distinct quantity or complexity to result in improvements in perching ability. Alternatively, the novelty hens may have been more accustomed to new environments, which may have resulted in them adjusting more rapidly to the layer pens rather than having skills specifically in perching. The novelty hens from the current study showed the greatest use of the large nest boxes in the layer pens across the flock cycle [30], indicating that these initial treatment differences in pen resource use persisted throughout lay. 

Birds reared with structural enrichments showed higher relative adrenal weights at the end of rearing. This could be indicative of a higher level of stress experienced during rear, resulting in higher production of corticosterone and consequently enlarged adrenal glands [31,32]. However, no other indicators of elevated stress in the structural pullets were specifically detected in the measurements taken (i.e., reduced body weight, increased fear, increased spleen or liver weights [32]), and differences were less than previously shown between healthy broilers and those with gait problems [31]). Thus, the differences, while statistically significant, may not have been biologically relevant (Table 2). Responses to chronic stress induced by the continuous delivery of corticosterone [33,34] or adrenocorticotropin in chickens [35] have included decreased relative weights of immune organs (bursa of Fabricius, thymus and spleen) and increased relative weights of the liver [33,34,35], due to an increase in liver lipids. However, previous comparisons on the effects of transport or social stress in layers have found conflicting increasing or decreasing effects on lymphoid organ weights [4,5]. Thus, changes in organ weights may not be sensitive enough to detect the effects of stressors. Further measurements on the structural pullets throughout the flock cycle, as part of the larger project, will reveal any potential negative impacts of this specific type of enrichment, although dissections on adult hens at 65 weeks of age did not show any differences in relative right adrenal weights between hens from all rearing treatments [36]. 

The CELL-DYN 3700 haematology showed some effects of rearing treatment and transport stress on blood parameters. However, the H/L ratio decreased following transport in the structural hens, the opposite direction to the predicted effect of stress on this measure [37]. The lymphocytes also increased following transport, again, the opposite direction to the documented effects of stress [37]. Thus, it is likely that the CELL-DYN 3700 cannot accurately differentiate all avian blood cell-types. Previous comparisons between manual counts and the CELL-DYN 3500 indicated very poor correlation between the manual and automated measures [38], although some authors found the CELL-DYN 3500 to be valuable in classifying corticosterone-mediated changes in heterophils [39]. Some authors have reported use of the CELL-DYN 3700 to categorise broiler blood types with or without worm parasites [40], however, some of the reported values in that study (e.g., MCH and MCHC) were also far outside of the normal chicken ranges as reported by [17]. 

Overall, it was expected that with the degree of variation between the rearing treatments, clearer differences between the pullets would have been found. However, it could be that measurements were made too early for differences to be detected, or that different behavioural or physiological assessments would have revealed variation. Assessments of skeletal structure, [3], for example, may have detected greater impacts of the structural enrichments. Measurements from some of the hens from the current study towards the end of the production cycle (64 weeks of age) did show effects of rearing treatments in the degree of plumage coverage, with less coverage in the control hens [36]. Based on the results from the pullets, it may be that in all rearing treatments there were sufficient resources supporting good pullet development. Any benefits of the enrichments might become obvious later in the production cycle, when hens are under increased physiological stress associated with egg production, outdoor access, social interaction and possibly age. 

## Figures and Tables

**Table 1:** The least squares means ± SEM of body weight in pullets from three rearing enrichment treatments (control, novelty, structural) as measured at four different age points (5, 8, 12, and 16 weeks of age).

Body Weight (kg)								
Treatment	5 Weeks	*n*	8 Weeks	*n*	12 Weeks	*n*	16 Weeks	*n*
Control	0.35 ± 0.004	15	0.70 ± 0.006 ^d^	567	1.12 ± 0.006 ^b,c^	548	1.37 ± 0.006 ^a^	532
Novelty	0.35 ± 0.004	15	0.69 ± 0.006 ^d^	551	1.09 ± 0.006 ^c^	547	1.36 ± 0.006 ^a^	519
Structural	0.35 ± 0.004	15	0.79 ± 0.006 ^d^	569	1.13 ± 0.006 ^b^	570	1.38 ± 0.006 ^a^	539

Birds were weighed in crate groups at five weeks of age but weighed individually from eight weeks of age onwards; sample size (*n*) is indicated for each age period. ^a,b,c,d^ Dissimilar letters indicate significant differences between rearing treatments across 8, 12, and 16 weeks of age.

**Table 2:** The mean percentage weights (± SEM) of organs relative to live body weight from 16-week-old pullets from three rearing enrichment treatments (control: *n* = 30, novelty: *n* = 30, structural: *n* = 29).

Treatment	% Liver Wgt	% Spleen Wgt	% Bursa Wgt	% Thymus Wgt	% Adrenal Wgt
Control	1.77 ± 0.03	0.16 ± 0.03	0.29 ± 0.01	0.21 ± 0.009	0.029 ± 0.0001 ^b^
Novelty	1.71 ± 0.03	0.16 ± 0.03	0.27 ± 0.01	0.21 ± 0.008	0.031 ± 0.001 ^b^
Structural	1.72 ± 0.03	0.16 ± 0.004	0.29 ± 0.02	0.21 ± 0.008	0.034 ± 0.001 ^a^

^a,b^ Dissimilar letters indicate significant differences between rearing treatments. Analyses were conducted on transformed proportional data, but raw percentages are presented.

**Table 3:** The mean ± SEM of latencies to struggle and vocalise (seconds), numbers of struggles and vocalisations, and plasma corticosterone concentrations during a manual restraint test for pullets from three rearing enrichment treatments (control: *n* = 30, novelty: *n* = 30, structural: *n* = 30).

Treatment	Latency Struggle (s)	Latency Vocalise (s)	Number of Struggles	Number of Vocals	Corticosterone ng/mL
Control	163.47 ± 18.96	31.87 ± 12.66	3.9 ± 1.30	58.69 ± 6.84	3.76 ± 0.28
Novelty	156.33 ± 19.53	32.37 ± 10.78	3.47 ± 0.70	71.66 ± 8.08	3.96 ± 0.34
Structural	190.07 ± 18.53	54.17 ± 16.68	2.47 ± 0.58	63.67 ± 13.17	3.46 ± 0.16

Analyses were conducted on transformed count data, but raw percentages are presented.

**Table 4:** The least squares means ± SEM concentrations of heterophils and lymphocytes and the heterophil/lymphocyte ratio for pullets from three rearing enrichment treatments (control: *n* = 30, novelty: *n* = 30, structural: *n* = 30) sampled before and after transport to the layer facility.

Variable	Transport	Control	Novelty	Structural
Heterophils 10^9^/L	Before	8.05 ± 0.43	8.58 ± 0.43	9.06 ± 0.43
	After	8.57 ± 0.43	8.18 ± 0.43	8.50 ± 0.43
Lymphocytes 10^9^/L	Before	21.91 ± 2.03	19.62 ± 2.03	15.48 ± 2.03
	After *	22.34 ± 2.03	23.40 ± 2.03	21.35 ± 2.03
H/L ratio	Before	0.52 ± 0.21 ^a,b^	0.73 ± 0.21 ^a,b^	1.48 ± 0.21 ^a^
	After	0.70 ± 0.21 ^a,b^	0.41 ± 0.21 ^b^	0.51 ± 0.21 ^b^

^a,b^ Dissimilar superscript letters indicate significant differences between rearing treatments and transport. * There was a significant main effect of transport on the concentrations of lymphocytes.
